# Fabrication of Laser-reduced Graphene Oxide in Liquid Nitrogen Environment

**DOI:** 10.1038/srep28913

**Published:** 2016-06-27

**Authors:** Y. C. Guan, Y. W. Fang, G. C. Lim, H. Y. Zheng, M. H. Hong

**Affiliations:** 1 Beihang University, 37 Xueyuan Road, Beijing, 100191, China; 2 Singapore Institute of Manufacturing Technology, 71 Nanyang Drive, 638075, Singapore; 3 National University of Singapore, 4 Engineering Drive 3, 117576, Singapore

## Abstract

Porous structure of reduced graphene oxide (rGO) plays an important role in developing flexible graphene-based devices. In this work, we report a novel methodology for reduction of freestanding graphite oxide (GO) sheet by picosecond pulse laser direct writing in liquid nitrogen. Non-agglomerate and porous structure of rGO is fabricated successfully due to frozen effect during laser processing. Compared with laser-irradiated rGO developed in N_2_ gas at ambient environment, the frozen rGO developed in liquid N_2_ shows better ordered structure with less defects, crack-free morphology as well as better electron supercapacitor performance including 50–60 Ω/sq in sheet electrical resistance. Mechanism of cryotemperature photoreduction GO is also discussed.

Reduction of graphite oxide (GO) has been well developed to produce graphene-based devices with novel electronic properties^1^,^2^,^3^,^4^. Various methods including chemical reaction, thermal treatment, photo-chemical reaction and laser reduction techniques have been applied to remove the oxygen species for reduction of GO, and the typical methods have been summarized in Table 1 to make a clear comparison of overall characteristics for GO reduction^5^,^6^,^7^,^8^,^9^,^10^,^11^,^12^. Laser reduction of GO has been recognized as a flexible way to produce reduced graphene oxide (rGO) for flexible patterning and integration of devices^5^,^12^,^13^,^14^,^15^,^16^,^17^,^18^,^19^,^20^,^21^,^22^,^23^,^24^,^25^,^26^. A variety of laser-irradiated GO methods have been reported, and most of them can be simply classified into two categories: The first is reduction of GO solution^12^,^13^,^14^,^15^, and the second is reduction and patterning of GO film with solid-state substrate^17^,^18^,^19^,^20^,^21^,^22^,^23^,^24^,^25^,^26^.

In order to improve mechanical properties of laser-irradiated rGO and avoid the limitation of temperature sensitive substrate for mass scalable devices, laser reduction of free standing GO sheet has attracted much attention. Koratka’s group reported CO_2_ laser-reduced freestanding GO sheet with thickness 10–20 μm as high-rate capable anodes for lithium-ion batteries, and found that the irradiated rGO with C/O ratio of 10.1 had defects including micrometer-scale pores, cracks, and intersheet voids^25^. Sokolov^26^ described reduction of freestanding GO sheet with thickness 5–8 μm to multilayer graphene using excimer laser irradiation in oxygen-free environments, and claimed that the best sheet resistance could be 100 Ω/sq and C/O ratio was 40. However, agglomeration of these rGO usually occurs because they are easily clustered at room temperature or above, which largely affects the electronic performance.

The aim of this paper is to provide a novel design for the reduction of freestanding GO sheet by one-stop photoreduction using a picosecond pulse laser. GO sheet was immersed in liquid nitrogen at −196 °C during laser irradiation to frozen the irradiated surface. The thickness of current freestanding GO sheet is measured as 20–30 μm, which is suitable for mechanically robust devices. The oxygen removal, ordered structure, and electrical performance of cryotemperature photoreduction of GO sheet were examined. Special attention is paid to understand the mechanism of cryotemperature photoreduction through comparison in normal N_2_ gas and liquid N_2_ conditions.

## Results

**Figure 1:**
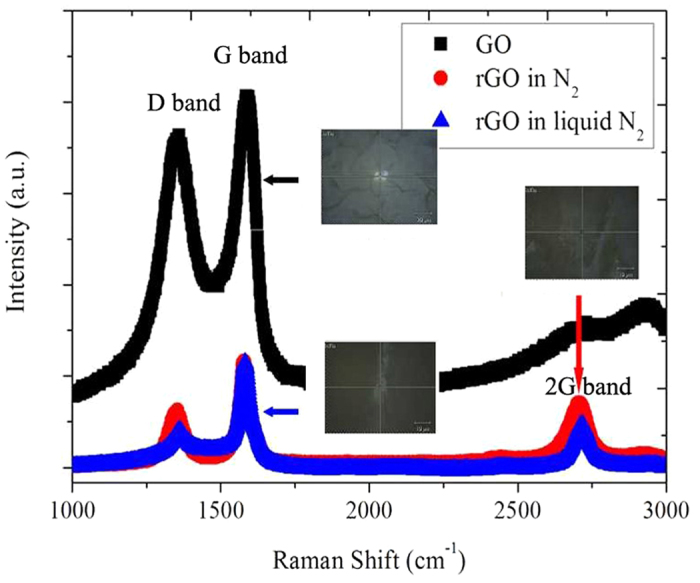
Raman spectra of as-received GO sheet and the generated rGO after laser irradiation in N_2_ gas/liquid N_2_.

Figure 1 shows Raman spectra of both GO and rGO before and after laser irradiation. Optical images show individual measured area of all samples, and the scale bar is 10 micron. It is known that the G and D bands result from E_2g_ phonon scattering of graphitic structure and vibrations of carbon atoms with dangling bonds in plane terminations of disordered graphite^12^, while the 2D-band is highly sensitive to stacking the graphene layers as the second order of the D-peak^19^. The presence of 2D-band at 2708 cm^−1^ indicates the considerable reduction of graphene oxides^19^. Quantitative results were summarized in Table 2. The overall changes in the D-band and G-band including narrowing and decrease in peak intensity as well as peak shift shows high ordered structure and production of graphene features in both laser irradiated rGO, which is in excellent agreement with previous findings^12^,^14^,^21^,^22^,^24^. More shift in the position for rGO in N_2_ gas compared to rGO in liquid N_2_ is mainly ascribed to the combined effect of remaining oxygen species after the reduction, defects associated with edge scattering in the rGO sheets as well as possible doping of rGO due to the presence of liquid N_2_ during laser process^15^,^27^.

The D/G intensity ratio (ID/IG) reduced significantly from 0.82 to 0.43 in N_2_ gas and 0.27 in liquid N_2_, respectively. This is supported by Mortazavi who reported the ratio of D to G-band (ID/IG) was about 0.5 based on nanosecond pulse laser ablation of graphite target^28^. The current ID/IG ratio is also comparable with laser reduction of GO film with solid substrate^19^,^25^. It should be noted that ID/IG ratios of rGO from liquid N_2_ are much lower than the value obtained from laser reduction of GO solution^14^,^15^,^16^,^17^,^18^. Such discrepancy as well as the difference between irradiation in N_2_ gas and in liquid N_2_ may correspond to photothermal and photochemical reduction between solid-state GO and GO solution during laser irradiation^12^. In addition, the change of ID/IG ratio offers a powerful evidence of N doping in rGO (N-rGO). According to the findings of Youn^27^ and Chen^29^, lower ID/IG ratio of N-rGO compared to that of rGO was attributed to the electron-donating capability of an N-heteroatom-induced graphitic degree decrease as well as nitrogen doping generates the decrease in the defects of high-surface-area N-rGO. Further investigation for the possible doping of rGO by XPS and XRD is needed.

It should be noted that temperature change caused by performing Raman measurement may occur at rGO sample surface, which can in turn alter the FWHM and frequency of the 2D and G bands^12^,^19^,^30^. In this work, however, we claim that the lattice change of rGO both in N_2_ gas and in liquid N_2_ is not remarkable, which might be due to the lower chance of having a significant radical temperature gradient effect on current rGO sample with thickness more than 20 micron^30^.

**Figure 2:**
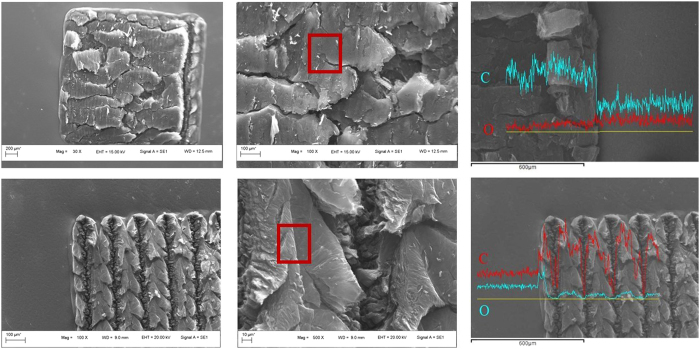
Microstrutrue features of rGO by laser direct writing. (**a**) SEM of laser induced rGO in N_2_ gas; (**b**) High magnification of (**a**); (**c**) EDS line scan of (**a**); (**d**) SEM of laser induced rGO in liquid N_2_; (**e**) High magnification of (**d**); (**f**) EDS line scan of (**b**).

**Figure 3:**
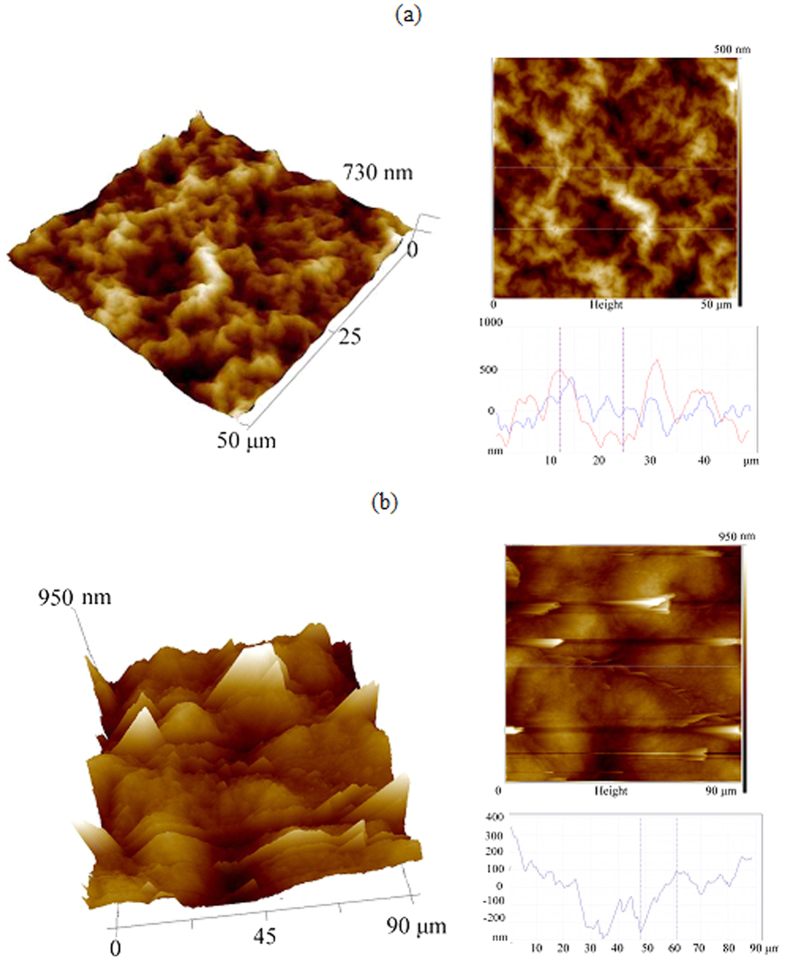
AFM 2D/3D analyses. (**a**) surface topography of rGO in N2 gas (**b**) surface topography of rGO in liquid N_2_.

To examine surface morphology and chemistry analysis of the irradiated rGO developed both in N_2_ gas and in liquid N_2_, all irradiated regions were characterized by SEM/EDS and AFM, as shown in Figs 2 and 3. Plate-shape and crack-like structure was observed in Fig. 2(a,b), indicating that GO sheet expansion took place during laser processing. No cracks, holes or voids was found in the microstructure after laser irradiation in liquid N_2_. Figure 2(d,e) show that periodic line-shape structure with average width of 100 μm was formed, and porous structure within line-shape between peaks and valleys was observed. Compared with the plate-shape structure in Fig. 2(b), the porous structure with clear edges among each layer in Fig. 2(e) are much looser, which might mainly due to frozen effect during laser irradiation at low temperature condition. The result is also consistent with what was previously reported about Raman analysis of high-surface-area N-rGO^27^. EDS line scan indicates distribution of main elements carbon and oxygen in the as received GO sheet and laser-irradiated rGO regions. Figure 2(c,f) show that carbon content was significant increased while oxygen content was decreased correspondingly at both top surfaces of rGO structure. It is interesting to find that both carbon and oxygen content drops at the valley of periodic structure, which may be related to detector limitation of EDS. Both AFM measurement areas were indicated in Fig. 2(b,e). The surface topograghy of these rGO structure was further measured in Fig. 3. It can be found that the surface in liquid N_2_ is rougher than that of N_2_ gas from both 3D images and 2D profilers, and the distance between the peaks and valleys was measured as around 600 nm. Compared with clustered structure in Fig. 3(a), the porous structure with clear edges could be found in Fig. 3(b).

Surface chemistry of both rGO was further analyzed by XPS, and the quantitative results were summarized in Table 3. It shows C-C, C-O and C = O reduced from 38.5%, 26.7%, 6.9% in GO to 67.4%, 19.5%, 5.1% in rGO of N_2_ gas and 75.3%, 12.3%, 3.6% in rGO of liquid N_2_, respectively, indicating that most of oxygen-containing functional groups have been removed after laser irradiation. The C/O ratio of as-received GO was calculated as 2.4, while the C/O ratio of the irradiated rGO rapidly increased as 9.1 in N_2_ gas and 10.3 in liquid N_2_. These results are consistent with the Raman analysis.

Therefore, we propose that the reduction of GO is improved in liquid N_2_ during laser irradiation, compared to that of N_2_ gas. The current C-C percentage is higher than nanosecond pulse laser irradiated in air with shorter wavelength, but lower than both nanosecond pulse laser irradiated in vacuum and femtosecond pulse laser irradiated in air with shorter wavelength, according to reduction degree of solid-state photoreduction in the literature^12^,^18^,^25^,^26^,^28^,^31^. The C/O ratio is lower than nanosecond pulse laser irradiated in air or in vacuum with shorter wavelength, but higher than that of CO_2_ laser. Correspondingly, we propose that cryotemperature photoreduction is not critical in restoring sp^2^ carbon network of GO by C-C peak and the C/O ratio, while laser wavelength plays more important role either by photoreduction or photochemical reduction.

**Figure 4:**
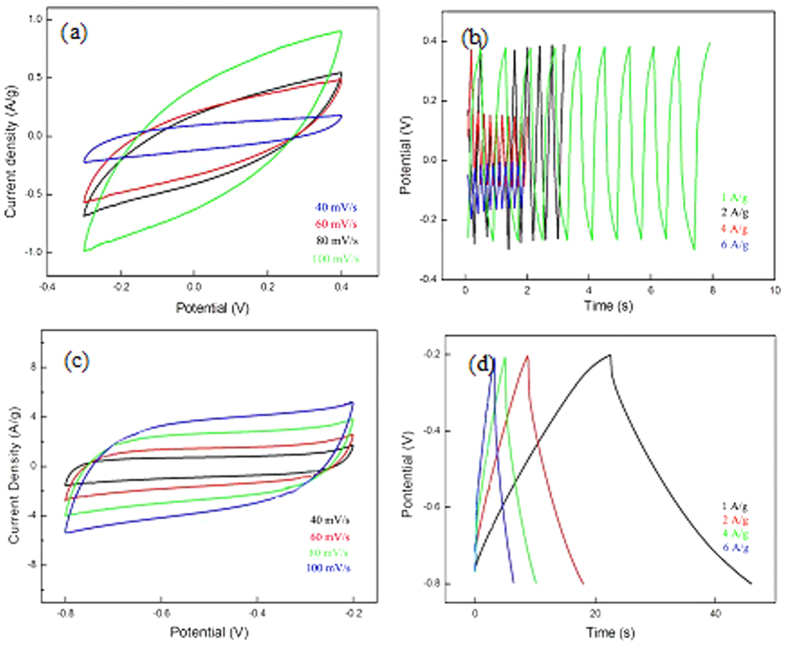
Electrochemical performance of rGO in both N_2_ gas and liquid N_2_. (**a**) CV profiles of rGO in N_2_ gas at different scan rates of 40, 60, 80, and 100 mV/s; (**b**) Galvanostatic charge/discharge curves of rGO in N_2_ gas at different current density of 1, 2, 4, 6 A/g; (**c**) CV profiles of rGO in liquid N_2_ at different scan rates of 40, 60, 80, and 100 mV/s; (**d**) Galvanostatic charge/discharge curves of rGO in liquid N_2_ at different current density of 1, 2, 4, 6 A/g.

The original graphite oxide sheet is completely insulating with sheet resistance values higher than >20 MΩ/sq using four-point probe measurements. After one-stop laser scanning in current work, the sheet resistance values of the irradiated rGO region were reduced to 10^4 ^Ω/sq in N_2_ gas and 50–60 Ω/sq in liquid N_2_, which is even lower than the best values as 100–500 Ω/sq reported by Sokolov^26^. The electrochemical behaviors of both rGO was further examined in Fig. 4. The scan range was optimized due to different current density and charge-discharge time. Figure 4(a,b) show poor performance for electrochemical performance of rGO sample in N_2_ gas. The specific capacitance (Csc) was measured to be 4 F/g at current density of 1 A/g, and it was reduced to 3.0 F/g, 1.71 F/g, 1.14 F/g, when the current density increased to 2 A/g, 4 A/g, and 6 A/g correspondingly. For rGO sample in liquid N_2_, the nearly rectangular CV shape at different scan rates indicates an enhanced electrochemical performance, which is indicative of potential capacitive behavior, as shown in Fig. 4(c). Figure 4(d) shows that the galvanostatic charge/discharge curves of rGO are linear and symmetric. Csc was measured to be 140 F/g at current density of 1 A/g. When the current density increased to 2, 4, and 6 A/g, Csc was reduced to 61.2, 33.2, 21.2 and 12.4 F/g, correspondingly. Our results need to be further improved including the optimization of laser parameters including energy density, overlapping ratio, repetition rate, scanning speed, etc.

## Discussion

During ultrashort pulse laser process, strong excitation of material surface leads to rapid ejection of species, such as electrons, photons or atoms, and the excited species will transfer energy to the lattice by typical electron-photon coupling^30^. Due to long wavelength irradiation, incident light at high fluence would create heat at the surface of GO sheet. It is generally accepted that plasma expansion driven by pressure and temperature difference above the irradiated surface is accompanied by emission of shock waves. However, heat conduction and GO sheet expansion was depressed in liquid nitrogen, thus plasma expansion and above surface plume interactions would be slightly different due to higher density gradient and more effective collisional cooling^32^. Furthermore, cryotemperature photoreduction of GO sheet and heat depression as well as frozen effect took place simultaneously in this work, thereby more accessible energy of laser imparts to the irradiated surface for ablation than thermal effect. The attractive force between the GO sheet and liquid nitrogen would arise from the nature of instantaneous fluctuating moments. Correspondingly, rapid evaporation occurred within the focal volume based on the intense molecular vibrational/rotational excitations^26^. The internal pressure between the layers exceeds up to overcome the lattice bonding energy within the freestanding GO sheet with thickness of 20–30 μm. This may explain the reason for frozen structure with crack free and porous structure of rGO development.

We suggest that depressed plasma expansion and frozen effect in liquid N_2_ accelerates the formation of energetic species in freestanding GO sheet by loosening the lattice bonds, resulting in more ordered state with fewer defects by photochemical desorption of oxygen groups and excellent sheet resistance and electrochemical properties of the irradiated rGO, compared with that of N_2_ gas background.

## Conclusions

We have proposed a novel design methodology for one-stop laser photoreduction of freestanding GO sheet. In specific, we have experimentally demonstrated the feasibility of cryotemperature rGO fabricated by picosecond pulse laser at liquid N_2_ condition, which enables promising electrical performance including the drop of sheet resistance by a factor of 10^4^ to 10^5^. Compared with plate-shape and crack-like structure of rGO developed in N_2_ gas, the frozen laser-irradiated rGO have crack-free porous structure mainly due to cryotemperature development and depressed thermal expansion, while the patterning parameters can be further tailored. Chemical analyses showed that the photochemical desorption of oxygen groups with better ordered structure and less defects on basis of 2D-band at 2708 cm^−1^, ID/IG ratio as 0.27, and C/O ratio as 10.3. On basis of widely used commercial picosecond pulse laser, this proof-of-concept process has the potential to effectively improve the development of manufacturing processes for flexible devices including roll-up displays, wearable devices, and implantable biodevices^1^,^12^.

## Methods

**Figure 5:**
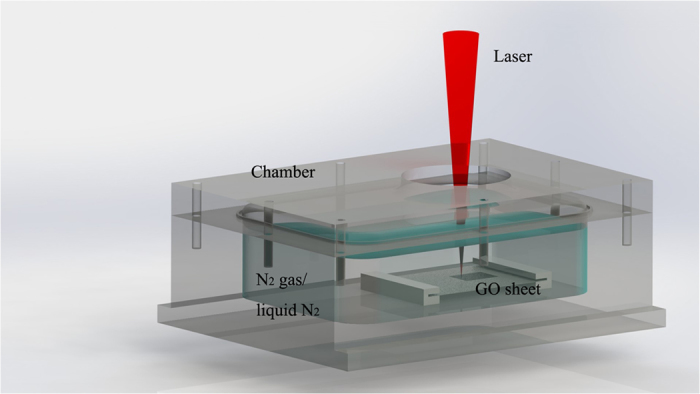
Schematic setup of direct laser writing for the reduction of GO.

In this work, the freestanding GO sheet was synthesized from modified chemical method^27^. Briefly, GO dispersion was exfoliated from graphite oxide through extensive ultrasonication, and placed in a casting mold made of polytetrafluoroethylene (PTFE). Correspondingly, the freestanding GO sheet was peeled off from the PTFE substrate after water was completely evaporated from the dispersion. The size of GO sheet in this work was 50 mm × 50 mm at thickness of 20–30 μm. A picosecond pulsed laser (wavelength 1064 nm, pulse duration 10 ps, repetition rate 100 000 Hz, spot size 30 μm) was used. The energy density was varied from 0.1 to 0.4 J/cm^2^, and scanning speed was changed in the range of 10–100 mm/s. The irradiated area was 10 × 10 mm^2^ in square using hatched scanning mode with 50% overlapping in the program. When laser was turned on, the specimen was placed in a well-sealed chamber filled with high purity nitrogen gas and liquid nitrogen, respectively, as shown in Fig. 5. No substrate under the GO sheet was provided. After laser exposure in liquid N_2_, the residual liquid N_2_ was evaporated at the room temperature.

Raman spectra were performed by a WITEC alpha 300 R Confocal Raman system in air ambient environment (532 nm excitation wavelength and 0.02 cm^−1^ spectral resolution). The Raman emission was collected by an Olympus 50× objective for better signal-to-noise ratio, and the excitation laser was set low power to prevent potential structure damages. Each measurement of Raman spectra was performed at 5 specimens for individual sample of GO, rGO in N_2_ gas, and rGO in liquid N_2_, respectively. Microstructural features of irradiated areas were investigated using Carl Zeiss EVO 50 scanning electron microscopy (SEM), equipped with an energy-dispersive X-ray spectrometer (EDS). The EDS measurements provided information on chemical composition. Surface topography of the irradiated areas was measured using Atomic Force Microscope (Bruker, Dimension FastScan).The non-contact mode is used to image 2D/3D profile and measure roughness of the textured surfaces. XPS analysis was carried out for the surface chemistry using Thermo Scientific Theta Probe XPS. Monochromatic Al Ka X-ray (hν = 1486.6 eV) was employed for analysis at an incident angle of 30° with respect to the surface normal. Photoelectrons are collected at a take-off angle of 50° with respect to the surface normal. The analyzed area is approximately 400 μm in diameter. Survey spectra and high-resolution spectra were acquired for surface composition analysis and chemical state identification, respectively. Sheet resistance measurements were performed by conventional four-point probe station using Keithley DMM source meter instrument at ambient conditions. Moreover, all electrochemical experiments were performed with CHI 660D electrochemical workstation. A three-electrode configuration was employed for all of the measurements with a platinum foil counter electrode and saturated calomel electrode (SCE) reference electrode. The working electrodes used was laser-reduced rGO, which was cut from the irradiated GO specimen. 0.5 M Na_2_SO_4_ was used as the electrolyte, and the potential scan was chosen in the range of −0.8 V and −0.2 V.

## Additional Information

**How to cite this article**: Guan, Y. C. *et al*. Fabrication of Laser-reduced Graphene Oxide in Liquid Nitrogen Environment. *Sci. Rep.* **6**, 28913; doi: 10.1038/srep28913 (2016).

## Figures and Tables

**Table 1:** Typical techniques for reduction of GO and their properties.

**Technique**	**Mechanism**	**Reduction degree**	**Conductivity or resistivity**	**Ref**
Chemical reaction	mixture acids	C/O:5.28 I_D_/I_G_:1.32	1251 S/m	5
	modified GO	I_D_/I_G_:1.36	1660 S/m	6
Thermal treatment	mixture GO dispersion	C/O: 6.59 I_D_/I_G_:1.35	5746 Ω/sq	7
	polycarbonate matrix	C/O:6.0	410 S/m	8
Photo-chemical reaction	UV lamp of GO suspension	C-OH, C = O, O = C-OH (%): 76%, 85% and 81% decrease	4.6 × 10^6 ^Ω/sq	9
	Hg lamp of GO sheet and chemical suspension	I_D_/I_G_:0.08 (single layer)	7.1 × 10^4 ^Ω/sq	10
Laser reduction	Excimer laser for GO solution	C/O: 40	100–500 Ω/sq	11
	Diode laser for GO sheet	C–C (%): 69.2	3830 S/m	12

**Table 2:** Parameters of GO and rGO derived from D, G, and 2D in Raman spectra.

	**D** (**Position**)	**D** (**FWHM**)	**G** (**Position**)	**G** (**FWHM**)	**ID/IG**	**2D** (**Position**)	**2D** (**FWHM**)
GO	1350 ± 2	180 ± 2	1585 ± 2	93 ± 1	0.82	–	–
rGO N_2_ gas	1364 ± 2	54 ± 1	1585 ± 1	42 ± 1	0.43	2718 ± 1	72 ± 1
rGO Liquid N_2_	1355 ± 2	46 ± 1	1578 ± 1	34 ± 1	0.27	2708 ± 1	67 ± 1

**Table 3:** Chemical compositions of GO and rGO derived from C 1 s spectra in XPS spectra (at.%).

	**C-C 285**.**0** (**eV**)	**C-O 286**.**5** (**eV**)	**C = O 288**.**7** (**eV**)
GO	38.5	26.7	6.9
rGO in N_2_ gas	67.4	19.5	5.1
rGO in liquid N_2_	75.3	12.3	3.6
